# Supplementary material for: Vitamin D and SARS-CoV2 infection, severity and mortality: A systematic review and meta-analysis
Source: PLoS One. 2022 Jul 6;17(7):e0268396. doi: 10.1371/journal.pone.0268396 (PMC9258852; doi:10.1371/journal.pone.0268396)
Supplement: S1 File — (DOCX) [file pone.0268396.s002.docx]

**Table S1.** PRISMA checklist (Moher et al. 2009).

| Section/topic | # | Checklist item | Reported on page # |
| --- | --- | --- | --- |
|  |  |  |  |
| TITLE |  |  |  |
| Title | 1 | Identify the report as a systematic review, meta-analysis, or both. | 1 |
| ABSTRACT |  |  |  |
| Structured Summary | 2 | Provide a structured summary including, as applicable: background; objectives; data sources; study eligibility criteria, participants, and interventions; study appraisal and synthesis methods; results; limitations; conclusions and implications of key findings; systematic review registration number. | 1 |
| INTRODUCTION |  |  |  |
| Rationale | 3 | Describe the rationale for the review in the context of what is already known. | 2 |
| Objectives | 4 | Provide an explicit statement of questions being addressed with reference to participants, interventions, comparisons, outcomes, and study design (PICOS). | 2 |
| METHODS |  |  |  |
| Protocol and registration | 5 | Indicate if a review protocol exists, if and where it can be accessed (e.g., Web address), and, if available, provide registration information including registration number. | 2 |
| Eligibility criteria | 6 | Specify study characteristics (e.g., PICOS, length of follow-up) and report characteristics (e.g., years considered, language, publication status) used as criteria for eligibility, giving rationale. | 3 |
| Information sources | 7 | Describe all information sources (e.g., databases with dates of coverage, contact with study authors to identify additional studies) in the search and date last searched. | 2-3 |
| Search | 8 | Present full electronic search strategy for at least one database, including any limits used, such that it could be repeated. | 2-3 |
| Study selection | 9 | State the process for selecting studies (i.e., screening, eligibility, included in systematic review, and, if applicable, included in the meta-analysis). | 4-5 |
| Data collection process | 10 | Describe method of data extraction from reports (e.g., piloted forms, independently, in duplicate) and any processes for obtaining and confirming data from investigators. | 3 |
| Data items | 11 | List and define all variables for which data were sought (e.g., PICOS, funding sources) and any assumptions and simplifications made. | 3 |
| Risk of bias in individual studies | 12 | Describe methods used for assessing risk of bias of individual studies (including specification of whether this was done at the study or outcome level), and how this information is to be used in any data synthesis. | 3 |
| Summary measures | 13 | State the principal summary measures (e.g., risk ratio, difference in means). | 4 |
| Synthesis of results | 14 | Describe the methods of handling data and combining results of studies, if done, including measures of consistency (e.g., I2) for each meta-analysis. | 4 |
| Risk of bias across studies | 15 | Specify any assessment of risk of bias that may affect the cumulative evidence (e.g., publication bias, selective reporting within studies). | 4 |
| Additional analyses | 16 | Describe methods of additional analyses (e.g., sensitivity or subgroup analyses, meta-regression), if done, indicating which were pre-specified. | 4 |
| RESULTS |  |  |  |
| Study selection | 17 | Give numbers of studies screened, assessed for eligibility, and included in the review, with reasons for exclusions at each stage, ideally with a flow diagram | 6 |
| Study characteristics | 18 | For each study, present characteristics for which data were extracted (e.g., study size, PICOS, follow-up period) and provide the citations. | STab1, STab2, STab3 |
| Risk of bias within studies | 19 | Present data on risk of bias of each study and, if available, any outcome level assessment (see item 12). | 3 |
| Results of individual studies | 20 | For all outcomes considered (benefits or harms), present, for each study: (a) simple summary data for each intervention group (b) effect estimates and confidence intervals, ideally with a forest plot. | 10-13 |
| Synthesis of results | 21 | Present results of each meta-analysis done, including confidence intervals and measures of consistency. | 5-9 |
| Risk of bias across studies | 22 | Present results of any assessment of risk of bias across studies (see Item 15). | 5-9 |
| Additional analysis | 23 | Give results of additional analyses, if done (e.g., sensitivity or subgroup analyses, meta-regression [see Item 16]). | 5-9 |
| DISCUSSION |  |  |  |
| Summary of evidence | 24 | Summarize the main findings including the strength of evidence for each main outcome; consider their relevance to key groups (e.g., healthcare providers, users, and policy makers). | 13-16 |
| Limitations | 25 | Discuss limitations at study and outcome level (e.g., risk of bias), and at review-level (e.g., incomplete retrieval of identified research, reporting bias). | 16 |
| Conclusions | 26 | Provide a general interpretation of the results in the context of other evidence, and implications for future research. | 17 |
| FUNDING |  |  |  |
| Funding | 27 | Describe sources of funding for the systematic review and other support (e.g., supply of data); role of funders for the systematic review. | 17-18 |
|  |  |  |  |

|  | **25OHD and Infection** | **25OHD and Covid-19**  **severity** | **Vitamin D supplementation and Covid-19 severity** | **25OHD and Covid-19 Mortality** | **Vitamin D supplementation and Covid-19**  **Mortality** |
| --- | --- | --- | --- | --- | --- |
| **Populations** | Healthy subjects | SARS-CoV2 positive subjects | SARS-CoV2 positive subjects | SARS-CoV2 positive subjects | SARS-CoV2 positive subjects |
| **Exposure/**  **Intervention** | Low 25OHD | Low 25OHD | Current vitamin D supplementation, any dose | Low 25OHD | Current vitamin D supplementation, any dose |
| **Comparison** | High 25OHD | High 25OHD | No current vitamin D supplementation | High 25OHD | No current vitamin D supplementation |
| **Outcome** | SARS-CoV2 infection | -Admission to hospital ICU  -Need for invasive mechanical ventilation;  -Intubation and hospital length of stay | -Admission to hospital ICU  -Need for invasive mechanical ventilation;  - Intubation and hospital length of stay | Covid-19 mortality | Covid-19  mortality |

**Table S2.** PICO/PECO strategy. PICO strategy summary table for research questions.

ICU: Intensive Care Unit.

**Table S3.** Quality of cross-sectional studies. Risk of bias and quality assessment of observational studies per outcome using the NewCastle Ottawa tool: Cross-sectional studies

| **Study** | **Representativeness of the sample** | **Sample size** | **Non-respondents** | **Assessment of exposure** | **Comparability of different outcome groups on the basis of design or analysis** | **Assessment of outcome** | **Statistical test** | **Score** | **Quality** | |  |
| --- | --- | --- | --- | --- | --- | --- | --- | --- | --- | --- | --- |
| ICU admission scenario | | | | | | | | | |  | |
| Jevalikar | - | * | - | * | ** | ** | * | 7 | Medium | |  |
| Maghbooli | * | - | - | * | - | * | * | 4 | Low | |  |
| ICU Admission and supplementation scenario | | | | | | | | | |  | |
| Jevalikar | - | * | - | * | ** | ** | * | 7 | Medium | |  |
| Mortality scenario | | | | | | | | | |  | |
| Jevalikar | - | * | - | * | ** | ** | * | 7 | Medium | |  |
| Luo | * | - | - | * | - | ** | * | 5 | Low | |  |
| Mortality and supplementation scenario | | | | | | | | | |  | |
| Jevalikar | - | * | - | * | ** | ** | * | 7 | Medium | |  |

**Table S4.** Quality of case-control studies. Risk of bias and quality assessment of observational studies per outcome using the NewCastle Ottawa tool: Case-control studies

| **Study** | **Adequate case definition** | **Representativeness of cases** | **Selection of controls** | **Definition of controls** | **Comparability of cases and controls on the basis of design or analysis** | **Assessment of exposure** | **Same method of ascertainment for cases and controls** | **Non-response rate** | **Score** | **Quality** |
| --- | --- | --- | --- | --- | --- | --- | --- | --- | --- | --- |
| **Infection scenario** | | | | | | | | | |  |
| Abdollahi | *** | * | * | - | ** | ***** | ***** | - | 7 | Medium |
| Ye | *** | * | * | * | - | ***** | **-** | - | 5 | Low |
| Hernandez | *** | * | * | - | * | ***** | **-** | - | 5 | Low |
| Lm | * | * | - | - | * | ***** | **-** | - | 4 | Low |
| Ferrari | * | * | - | * | ** | ***** | ***** | - | 7 | Medium |
| ICU scenario | | | | | | | | | |  |
| Hernandez | * | * | - | * | ** | ***** | ***** | - | 7 | Medium |
|  |  |  |  |  | ICU and supplementation scenario |  |  |  |  |  |
| Hernandez | * | * | - | * | * | ***** | ***** | * | 7 | Medium |
|  |  |  |  |  | Mortality scenario |  |  |  |  |  |
| Hernandez | * | * | - | * | ** | ***** | ***** | - | 7 | Medium |
| Tort | * | * | * | * | ** | ***** | ***** | - | 8 | Medium |
|  |  |  |  |  | Mortality and supplementation scenario |  |  |  |  |  |
| Hernandez | * | * | - | * | * | ***** | ***** | * | 7 | Medium |

**Table S5.** Quality of cohort studies. Risk of bias and quality assessment of observational studies per outcome using the NewCastle Ottawa tool: Cohort studies

| **Study** | **Representativeness exposed cohort** | **Selection of non- exposed cohort** | **Ascertainment of exposure** | **Absence of outcome of interest at start of study** | **Comparability of cohorts on the basis of design or analysis** | **Assessment of outcome** | **Follow-up enough for outcome to occur** | **Adequacy of follow up of cohorts** | | **Score** | **Quality** |
| --- | --- | --- | --- | --- | --- | --- | --- | --- | --- | --- | --- |
| Infection scenario | | | | | | | | | | |  |
| Melzer | ***** | ***** | ***** | ***** | ****** | ***** | ***** | **-** | | 8 | Medium |
| Merzon | ***** | ***** | **-** | ***** | ****** | ***** | **-** | **-** | | 6 | Low |
| Kaufman | ***** | ***** | **-** | ***** | ****** | ***** | **-** | **-** | | 6 | Low |
| ICU admission | | | | | | | | | | |  |
| Mendy | ***** | ***** | ***** | ***** | ****** | ***** | **-** | **-** | | 7 | Medium |
| Radjukovic | **-** | ***** | **-** | ***** | ***** | ***** | **-** | - | | 4 | Low |
| Faul | **-** | ***** | **-** | ***** | ***** | ***** | **-** | - | | 4 | Low |
| Panagiotou | **-** | ***** | **-** | ***** | ***** | ***** | **-** | - | | 4 | Low |
| Alguwaihes | **-** | **-** | **-** | ***** | ***** | ***** | **-** | - | | 3 | Low |
| Carpagnano | ***** | ***** | ***** | ***** | ****** | ***** | **-** | - | | 7 | Medium |
| Walk | ***** | ***** | ***** | ***** | ****** | ***** | **-** | - | | 7 | Medium |
| Lau | ***** | ***** | ***** | ***** | ****** | ***** | **-** | - | | 7 | Medium |
| Jain | **-** | ***** | ***** | ***** | **-** | ***** | **-** | - | | 4 | Low |
| Gavioli | ***** | ***** | ***** | ***** | ****** | ***** | **-** | - | | 7 | Medium |
| Lohia | ***** | ***** | ***** | ***** | ****** | ***** | **-** | - | | 7 | Medium |
| Orchard | ***** | ***** | ***** | ***** | ****** | ***** | **-** | - | | 7 | Medium |
| Baktash | ***** | ***** | ***** | ***** | ****** | ***** | **-** | - | | 7 | Medium |
| ICU admission and supplementation scenario | | | | | | | | | | |  |
| Tan | ***** | ***** | ***** | ***** | ****** | ***** | **-** | **-** | | 7 | Medium |
| Giannini | ***** | ***** | ***** | ***** | ****** | ***** | **-** | **-** | | 7 | Medium |
| Mortality scenario | | | | | | | | | | |  |
| De Smet | ***** | ***** | ***** | ***** | ****** | ***** | **-** | | **-** | 7 | Medium |
| Mendy | ***** | ***** | ***** | ***** | ****** | ***** | **-** | | **-** | 7 | Medium |
| Radjukovic | **-** | ***** | **-** | ***** | ***** | ***** | **-** | | **-** | 4 | Low |
| Karonova | ***** | **-** | **-** | ***** | **-** | ***** | **-** | | **-** | 3 | Low |
| Abrishami | **-** | ***** | ***** | ***** | ***** | ***** | **-** | | ***** | 6 | Low |
| Alguwaihes | ***** | **-** | **-** | ***** | ****** | ***** | **-** | | **-** | 5 | Low |
| Carpagnano | ***** | ***** | ***** | ***** | ***** | ***** | **-** | | **-** | 6 | Low |
| Vassiliou | **-** | ***** | ***** | ***** | **-** | ***** | ***** | | ***** | 6 | Low |
| Jain | **-** | ***** | ***** | ***** | **-** | ***** | ***** | | ***** | 6 | Low |
| Bennouar | **-** | ***** | ***** | ***** | **-** | ***** | **-** | | **-** | 4 | Low |
| Gavioli | ***** | ***** | ***** | ***** | ****** | ***** | **-** | | **-** | 7 | Medium |
| Lohia | ***** | ***** | ***** | ***** | ****** | ***** | **-** | | **-** | 7 | Medium |
| Orchard | ***** | ***** | ***** | ***** | ****** | ***** | **-** | | **-** | 7 | Medium |
| Barassi | ***** | ***** | ***** | ***** | ****** | ***** | **-** | | **-** | 7 | Medium |
| Baktash | ***** | ***** | ***** | ***** | ****** | ***** | **-** | | **-** | 7 | Medium |
| Mortality and supplementation scenario | | | | | | | | | | |  |
| Annweiler | ***** | ***** | ***** | ***** | ****** | ***** | **-** | **-** | | 7 | Medium |
| Cangiano | ***** | ***** | ***** | ***** | ****** | ***** | **-** | **-** | | 7 | Medium |
| Ling | ***** | ***** | ***** | ***** | ****** | ***** | **-** | **-** | | 7 | Medium |

| TableS 6. Quality of trial. Quality assessment of included articles according to Cochrane tool | | | | | | | |
| --- | --- | --- | --- | --- | --- | --- | --- |
| Study | **Random sequence generation** | **Allocation concealment** | **Blinding of participants and personnel** | **Blinding of outcome assessment** | **Incomplete outcome data** | **Selective reporting** | **Other bias** |
| Castillo | L | U | L | L | L | L | L |
| Murai | L | U | L | L | L | L | U |
| L: low risk, H: high risk, U: unclear risk | | | | | | | |

**Table S7. Infection scenario.** Table of included studies evaluating the association between high vs low 25OHD and risk of Sars-Cov2 infection.

| **FA, (Country)** | **Accrual start** | **Accrual stop** | **Study design** | **Age*** | **N^** | **% male** | **Thresholds of VD leavels** | **Time of VD measurement** | **Adjustments** |
| --- | --- | --- | --- | --- | --- | --- | --- | --- | --- |
| Abdollahi, (Iran) | 20/02/2020 | 20/04/2020 | CC | 48 | 402 | 33 | 30 ng/mL | Tested at admission to hospital |  |
| Ferrari, (Italy) | 20/02/2020 | 07/04/2020 | CC | 61.02 | 347 | 55 |  | Tested at admission to hospital |  |
| Hernandez, (Spain) | 10/03/2020 | 31/03/2020 | CC | 61 | 394 | 62 | 20 ng/mL | Tested at admission to hospital |  |
| Im, (South Korea) | 01/02/2020 | 01/06/2020 | CC | 52.2 | 200 | 58 | 20 ng/mL | Tested within 7 days of admission |  |
| Kaufman, (US) | 09/03/2020 | 19/06/2020 | Ret. CO | 54 | 191779 | 32 | 20 ng/mL | Tested in the previous year, adjusted for season |  |
| Melzer, (US) | 03/03/2020 | 10/04/2020 | Ret. CO | 49.2 | 489 | 25 |  | 1,25(OH)­2D or 25(OH)D from one year to 14 days before the infection | Age, gender, comorbidities, BMI, |
| Merzon, (Israel) | 01/02/2020 | 30/04/2020 | Ret. CO | 35.6 | 7807 | 49 | 30 ng/mL | One previous 25(OH)D test | Age, gender, comorbidities, BMI, smoke |
| Ye, (China) | 16/02/2020 | 16/03/2020 | CC | 43 | 142 | 37 | 50 ng/mL | Tested at admission to hospital |  |

*Age (mean); ^total number of patients enrolled; CC: case-control study, Ret. Co: retrospective cohort study; FA: First author: Adjustments: Adjustment of risk estimates for confounders.

**Table S8. Vitamin D levels.**Table of included studies evaluating the association between 25(OH)D at baseline and risk of severity and mortality.

| **FA, country** | **Accrual start** | **Accrual stop** | **Study design** | **Outcome** | **Age*** | **N^** | **% male** | **Thresholds of VD leavels** | **Time of VD measurement** | **Adjustments** |
| --- | --- | --- | --- | --- | --- | --- | --- | --- | --- | --- |
| Abrishami (Iran) | 28/02/2020 | 19/04/2020 | Pro. CO | Mortality | 55.18 | 73 | 64 | 25 ng/mL | Admission to hospital | Age, gender and comorbidities |
| Alguwaihes (Saudi Arabia) | 01/05/2020 | 30/07/2020 | Ret. CO | Mortality and admission to ICU | 55 | 439 | 68 | 5 ng/mL | Admission to hospital | Age, gender and BMI |
| Baktash (UK) | 01/03/2020 | 30/04/2020 | Pro. CO | Mortality and ventilation requirement |  | 70 | 60 | 30 ng/mL | Admission to hospital |  |
| Barassi (Italy) | 08/04/2020 | 25/05/2020 | Pro. CO | Mortality | 61 | 118 | 63 | 20 ng/mL | Admission to hospital |  |
| Bennouar (Algeria) | 06/07/2020 | 15/08/2020 | Pro. CO | Mortality | 62.3 | 120 | 69 | <10 ng/mL (low) and >30 ng/mL (high) | Admission to hospital | Age, gender and other adj |
| Carpagnano (Italy) | 11/03/2020 | 30/04/2020 | Pro. CO | Mortality and admission to ICU | 65 | 42 | 71 | 10 ng/mL | First 12h following ICU admission |  |
| De Smet (Belgium) | 01/03/2020 | 07/04/2020 | Pro. CO | Mortality | 69 | 186 | 59 | 20 ng/mL | Admission and within 24 hours from chest CT staging | Age, gender, comorbidities, diabetes and other adj |
| Faul (Ireland) | 01/03/2020 | 30/03/2020 | Pro. CO | Severity (Intubation) |  | 33 | 100 | 30 ng/mL | Admission to hospital |  |
| Gavioli (New York) | 01/03/2020 | 08/05/2020 | Ret. CO | Mortality and Oxigen support | 67 | 437 | 48 | 20 ng/mL | Within 3 months prior to the SARS-CoV-2 test, or within their admission hospital labs | Age, gender, comorbidities, BMI, ethnicity and other adj |
| Hernandez (Spain) | 10/03/2020 | 31/03/2020 | CC | Mortality and admission to ICU |  | 216 | 60 | 20 ng/mL | Admission to hospital |  |
| Jain (India) | 05/06/2020 | 20/07/2020 | Pro. CO | Mortality and admission to ICU |  | 154 | 62 | 20 ng/mL | Admission to hospital |  |
| Jevalikar (India) | 09/07/2020 | 08/08/2020 | Cross-sect. | Mortality and Admission to ICU | 54 | 409 | 69 | 20 ng/mL | Admission to hospital |  |
| Karonova (Russia) | -. | - | Ret. CO | Mortality | 53.2 | 80 | 54 | 20 ng/mL | Admission to hospital |  |
| Lau (US) | 27/03/2020 | 21/04/2020 | Ret. CO | Severity (admission to ICU) | 65,2 | 20 | 45 | 30 ng/mL | Admission to hospital |  |
| Lohia (USA) | 10/03/2020 | 30/06/2020 | Ret. CO | Mortality and admission to ICU | 63.8 | 270 | 43 | <20 ng/mL (low) and >30 ng/mL (high) | One year before | Age, gender, comorbidities and BMI |
| Luo (China) | 27/02/2020 | 21/03/2020 | Cross-sect. | Mortality | 56 | 335 | 44 | 30 ng/mL | Admission to hospital |  |
| Maghbooli (Iran) |  | 01/05/2020 | Cross-sect. | Severity (admission to ICU) | 58,72 | 235 | 61 | 30 ng/mL | Admission to hospital or soon thereafter |  |
| Mendy (US) | 13/03/2020 | 31/05/2020 | Pro. CO | Mortality and admission to ICU | 49.5 | 689 | 53 |  | Admission to hospital | Age, gender, ethnicity and other adj |
| Orchard (UK) | 19/03/2020 | 15/06/2020 | Ret. CO | Mortality and ventilation requirement | 60 | 50 | 56 | 50 ng/mL | Admission to hospital |  |
| Panagiotou (UK) |  |  | Ret. CO | Severity (admission to ICU) |  | 134 | 55 | 50 ng/mL | Admission to hospital |  |
| Radjukovic (Germany) | 18/03/2020 | 18/06/2020 | Ret. CO | Mortality and ventilation requirement | 60 | 185 | 51 | 12 ng/mL | Admission to hospital | Age, gender and comorbidities |
| Tort (Mexico) | 01/03/2020 | 30/04/2020 | CC | Mortality |  | 172 | 77 | 8 ng/mL | Admission to hospital |  |
| Vassiliou (Grece) | 22/03/2020 | 03/08/2020 | Pro. CO | Mortality | 65 | 30 | 80 | 15.2 ng/mL | Admission to ICU |  |
| Walk (Netherland) | 01/03/2020 | 30/04/2020 | Pro. CO | Severity (Intubation) | 68 | 133 | 69 | <25 ng/mL (low) and >50 ng/mL (high) | Admission to hospital |  |

*Age (mean); ^total number of patients enrolled; CC: case-control study, Ret. Co: retrospective cohort study; Pro. Co: prospective cohort study; FA: First author: Adjustments: Adjustment of risk estimates for confounders.

**Table S9. Supplementation.** Table of included studies evaluating the association between vitamin D supplementation and risk of severity and mortality.

| **FA, country** | **Accrual start** | **Accrual stop** | **Study design** | **Outcome** | **Age*** | **N^** | **% male** | **Supplementation** | **Adjustments** |
| --- | --- | --- | --- | --- | --- | --- | --- | --- | --- |
| Annweiler (France) | 01/03/2020 | 30/05/2020 | Ret. CO | Mortality | 88 | 66 | 51 | Group 1: 50.000 UI Cholecalciferol monthly or 80.000 - 10.000 UI every 2-3 months; Group 2: one dose- 80.000 UI Cholecalciferol within a few hours of COVID-19 diagnosis; | Age, gender, comorbidities, hypertension and other adj |
| Cangiano (Italy) | 01/03/2020 | 30/04/2020 | Ret. CO | Mortality | 90 | 98 | 29 | Cholecalciferol 25.000 UI every 2 weeks |  |
| Castillo (Spain) | - | - | RCT | Mortality and ICU admission | 53 | 76 | 59 | Oral calcifediol, 0,532 mg on day 1; 0,266 mg on day 3 and day 7; then weekly until discharge | Hypertension and diabetes |
| Giannini (Italy) | 15/03/2020 | 30/04/2020 | Pro. CO | ICU admission | 74 | 91 | 55 | 400.000 UI, bolus oral cholecalciferol (200,000 IU administered in two consecutive days) | Comorbidities |
| Hernandez (Spain) | 10/03/2020 | 31/03/2020 | CC | Mortality and ICU admission |  | 216 | 60 | 11 patients were taking cholecalciferol, 25000 IU/monthly in 10 cases, and 5600 IU/weekly in 1, and 8 patients were on calcifediol, 0.266 mg/monthly |  |
| Jevalikar (India) | 09/07/2020 | 08/08/2020 | Cross-sect. | Mortality and ICU admission | 54 | 409 | 69 | 60.000 UI (median) Cholecalciferol until discharge or death |  |
| Ling (UK) | 27/01/2020 | 05/08/2020 | Ret. CO | Mortality | 74 | 444 | 55 | Cholecalciferol high-dose booster therapy ≥280,000 IU in a time period of up to 7 weeks for patients deficient or insufficient | Age, gender, comorbidities, diabetes, ethnicity and other adj |
| Ling (UK) | 27/01/2020 | 05/08/2020 | Ret. CO | Mortality | - | 540 | 57,8 | Cholecalciferol high-dose booster therapy ≥280,000 IU in a time period of up to 7 weeks for patients deficient or insufficient | Age, gender, comorbidities, diabetes, ethnicity and other adj |
| Murai (Brazil) | 02/06/2020 | 27/08/2020 | RCT | Mortality and ICU admission | 56,2 | 237 | 56 | 200,000 IU Cholecalciferol on day 1 |  |
| Tan (Singapore) | 15/01/2020 | 15/04/2020 | Ret. CO | Requiring oxygen therapy (includ-ing ICU support) | - | 43 | 61 | 1000 UI Cholecalciferol daily upon admission | Age |

*Age (mean); ^total number of patients enrolled; CC: case-control study, Ret. Co: retrospective cohort study; Pro. Co: prospective cohort study; FA: First author: Adjustments: Adjustment of risk estimates for confounders.

**Table S10.** Infection analysis. Subgroup analysis related to association between baseline serum vitamin D levels and risk of SARS-CoV2 infection scenario

|  | N | RR (95% CI) | $I^{2}$ | P.value* |
| --- | --- | --- | --- | --- |
| Geographic area |  |  |  | 0.73 |
| Asia | 4 | 2.26 (1.49-3.43) | 63.76% |  |
| Other (Europe=1 and USA=2) | 4 | 2.07 (1.15-3.74) | 91.67% |  |
| Quality  Low  Medium | 5  3 | 2.55 (1.52-4.27)  1.77 (1.35-2.32) | 92%  0% | 0.3 |

N=number of studies; RR= relative risk; CI=confidence interval;*P value for the difference between groups.

**Table S11.** Severity and vitamin D levels. Subgroup analysis related to Association between baseline serum vitamin D levels and severity of Covid-19 scenario

|  | N | RR (95% CI) | $I^{2}$ | P.value* |
| --- | --- | --- | --- | --- |
| Type study |  |  |  | 0.9 |
| Prospective | 8 | 2.59 (1.04-6.46) | 86.85% |  |
| Retrospective | 8 | 2.22 (1.66-2.96) | 0% |  |
| Comorbidities |  |  |  | 0.89 |
| Adjust | 3 | 2.19 (1.56-3.09) | 0% |  |
| No adjust | 13 | 2.40 (1.37-4.22) | 73.74% |  |
| BMI |  |  |  | 0.76 |
| Adjust | 3 | 2.06 (1.45-2.92) | 0% |  |
| No adjust | 13 | 2.54 (1.43-4.53) | 75.62% |  |
| Season  Summer  Other | 4  12 | 3.53 (0.53-23.48)  2.22 (1.73-2.86) | 94.79%  0% | 0.82 |
| Geographic area  Asia  USA  Europe | 4  4  8 | 3.54 (0.53-23.61)  2.15 (1.57-2.94)  2.29 (1.48-3.54) | 94.23%  0%  12.99% | 0.97 |
| Severity type  ICU admission  Meccanical ventilation  Other | 10  3  3 | 2.5 (1.26-4.96)  3.59 (1.65-7.83)  1.96 (1.15-3.34) | 81.61%  0%  30.61% | 0.70 |
| Quality  Low  Medium | 6  10 | 4.58 (1.72-12.23)  1.64 (1.11-2.42) | 79.49%  49.37% | 0.02 |

N=number of studies; RR= relative risk; CI=confidence interval;*P value for the difference between groups.

**Table S12.** Severity and supplementation. Subgroup analysis related to Association between vitamin D supplementation and severity of Covid-19 scenario

|  | N | RR (95% CI) | $I^{2}$ | P.value* |
| --- | --- | --- | --- | --- |
| Season |  |  |  | 0.03 |
| Summer | 2 | 0.67 (0.40-1.12) | 0% |  |
| Other | 4 | 0.20 (0.07-0.56) | 44.37% |  |
| Geographic area  Europe  Other (USA=1 and Asia=2) | 3  3 | 0.17 (0.04-0.79)  0.59 (0.36-0.96) | 61.28%  0% | 0.11 |
| Severity type  ICU admission  Other | 4  2 | 0.30 (0.09-1.05)  0.37 (0.17-0.80) | 77.84%  0% | 0.43 |
| Study type  RCT  Observational | 2  4 | 0.18 (0.08-3.79)  0.44 (0.25-0.75) | 86.07%  0% | 0.61 |
| Quality  High  Medium | 2  4 | 0.18 (0.008-3.79)  0.44 (0.25-0.75) | 86.08%  0% | 0.61 |
| Initial bolus  Yes  No | 3  3 | 0.53 (0.31-0.90)  0.18 (0.03-1.06) | 7.5%  70.5% | 0.46 |
| Initial dose  Regular  High | 1  2 | 0.19 (0.04-0.93)  0.60 (0.35-1.03) | -  0% | 0.18 |
| Monthly dose  Regular  High | 3  2 | 0.20 (0.04-1.01)  0.60 (0.35-1.03) | 73%  0% | 0.28 |

N=number of studies; RR= relative risk; CI=confidence interval;*P value for the difference between groups.

**Table S13.** Mortality and vitamin D levels. Subgroup analysis related to Association between baseline serum vitamin D levels and mortality of Covid-19 scenario

|  | N | RR (95% CI) | $I^{2}$ | P.value* |
| --- | --- | --- | --- | --- |
| Age and gender |  |  |  | 0.36 |
| Adjust | 7 | 3.06 (1.41-6.62) | 66.01% |  |
| No adjust | 12 | 1.95 (1.05-3.61) | 59.70% |  |
| Comorbidities |  |  |  | 0.74 |
| Adjust | 4 | 2.83 (0.94-8.48) | 70.13% |  |
| No adjust | 15 | 2.26 (1.30-3.92) | 62.89% |  |
| Season  Summer  Other | 6  13 | 2.88 (0.87-9.59)  2.14 (1.32-3.47) | 80.54%  45.82% | 0.85 |
| Geographic area  Europe  Asia  USA  Other | 8  6  4  1 | 2.56 (1.29-5.10)  3.15 (1.09-9.08)  1.28 (0.65-2.51)  - | 24.96%  64.48%  71.42%  - | 0.18 |
| Quality  Low  Medium | 9  10 | 6.18 (3.52-10.85)  1.29 (0.79-2.12) | 0%  57.49% | <0.0001 |

N=number of studies; RR= relative risk; CI=confidence interval;*P value for the difference between groups.

**Table S14.** Mortality and supplementation. Subgroup analysis related to Association between vitamin D supplementation and mortality of Covid-19 scenario

|  | N | RR (95% CI) | $I^{2}$ | P.value* |
| --- | --- | --- | --- | --- |
| Season |  |  |  | 0.38 |
| Summer | 4 | 0.44 (0.18-1.06) | 66.94% |  |
| Other | 4 | 0.24 (0.07-0.82) | 46.01% |  |
| Geographic area  Europe  Other (Asia=1 and USA=1) | 6  2 | 0.29 (0.18-0.45)  0.66 (0.08-5.25) | 0%  65.22% | 0.02 |
| Study type  RCT  Observational | 2  6 | 0.67 (0.06-6.90)  0.29 (0.18-0.45) | 55.9%  0% | 0.01 |
| Type study  Prospective  Retrospective | 4  4 | 0.36 (0.09-1.40)  0.31 (0.19-0.51) | 60.79%  0% | 0.63 |
| Quality  High  Medium | 2  6 | 0.67 (0.06-6.90)  0.29 (0.18-0.45) | 55.9%  0% | 0.01 |
| Initial bolus  Yes  No | 5  3 | 0.33 (0.14-0.78)  0.40 (0.09-1.77) | 70%  28.2% | 0.79 |
| Initial dose  Regular  High | 2  3 | 0.14 (0.04-0.41)  0.49 (0.18-1.37) | 0%  77% | 0.09 |
| Monthly dose  Regular  High | 5  3 | 0.23 (0.08-0.65)  0.49 (0.18-1.37) | 33%  77% | 0.09 |

N=number of studies; RR= relative risk; CI=confidence interval;*P value for the difference between groups.


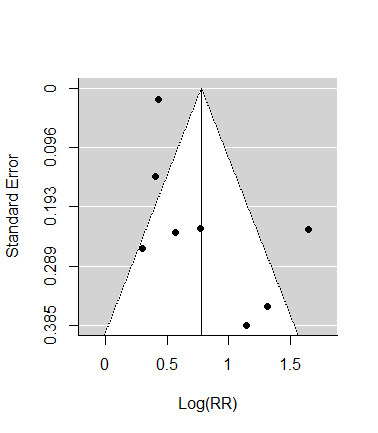


**Supplementary figure 1.** Funnel plot of infection scenario. Funnel plot related to association between baseline serum vitamin D levels and risk of SARS-CoV-2 infection scenario


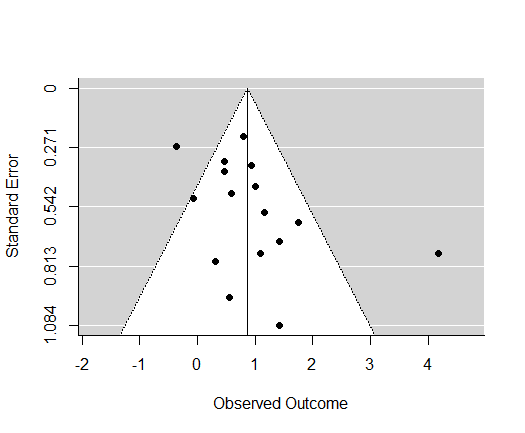


**Supplementary figure 2.** Funnel plot of severity and vitamin D levels scenario. Funnel plot related to association between baseline serum vitamin D levels and severity of Covid-19 scenario


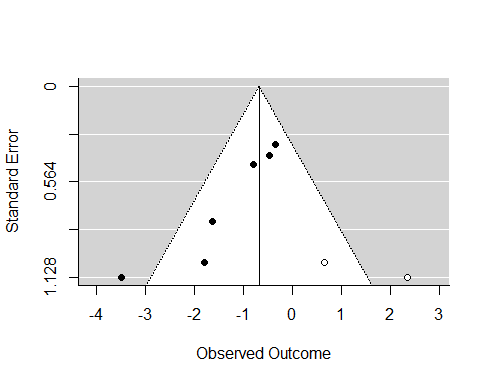


**Supplementary figure 3.** Funnel plot of severity and supplementations scenario. Funnel plot related to association between vitamin D supplementation and the severity of Covid-19 scenario


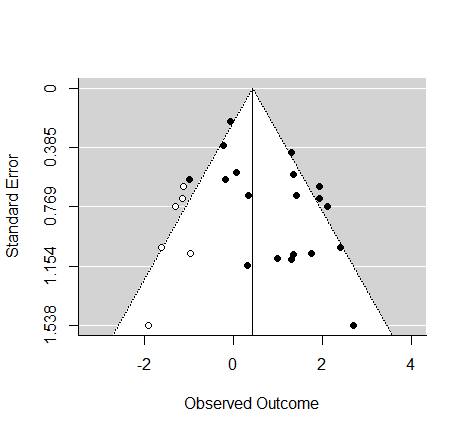


**Supplementary figure 4.** Funnel plot of mortality and vitamin D levels. Funnel plot related to association between baseline serum vitamin D levels and mortality in patients with Covid-19 scenario


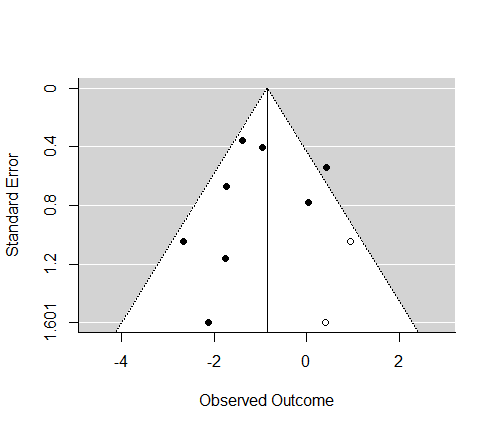


**Supplementary figure 5.** Funnel plot of mortality and supplementation scenario. Funnel plot related to association between vitamin D supplementation and mortality in patients with Covid-19 scenario
